# ﻿A new species of *Amorphophallus* (Araceae) from northeastern Thailand

**DOI:** 10.3897/phytokeys.229.106466

**Published:** 2023-07-13

**Authors:** Wilawan Promprom, Wannachai Chatan, Pattana Pasorn, Narueset Prasertsri, Thinnakon Angkahad

**Affiliations:** 1 Department of Biology, Faculty of Science, Mahasarakham University, Kantharawichai District, Mahasarakham Province, 44150, Thailand Mahasarakham University Maha Sarakham Thailand; 2 Plant and Innovation Research Unit, Mahasarakham University, Mahasarakham, 44150, Thailand Mahasarakham University Maha Sarakham Thailand; 3 Walai Rukhavej Botanical Research Institute, and Center of Excellence in Biodiversity Research, Mahasarakham University, Maha Sarakham 44150, Thailand Mahasarakham University Maha Sarakham Thailand; 4 Department of Geo-Informatics Technology, Faculty of Informatics, Mahasarakham University, Mahasarakham 44150, Thailand Mahasarakham University Maha Sarakham Thailand

**Keywords:** *Amorphophallussakonnakhonensis*, Araceae, aroid, plant diversity, plant taxonomy

## Abstract

*Amorphophallussakonnakhonensis* Chatan & Promprom, a new species from northeastern Thailand, is described and illustrated. The new species is most similar to *A.harmandii* Engl. & Gehrm. and *A.linearis* Gagnep., but it is distinguished by the combination of characters as follows: clear differences with *A.harmandii* are shorter style; disc-like, slightly smooth surface, concave centre, ca. 0.2 × 0.1 mm stigma; slightly cylindrical, slightly narrower upper part of staminate flower zone; slightly cylindrical to elongate-fusiform, erect or slightly erect, creamy white appendix. The clearly distinct morphology with *A.linearis* are disc-like, slightly smooth surface, concave centre, ca. 0.2 × 0.1 mm stigma; elliptic or obovate leaflet; 1–3 cm long, creamy white appendix. The preliminary conservation status was assessed, and the distinct characteristics of similar species were discussed.

## ﻿Introduction

*Amorphophallus* Blume ex Decne. (Decaisne 1834) is a genus in the family Araceae that contains about 200–250 species (Hetterscheid 2012; Claudel et al. 2017; POWO 2023) and which is distributed from tropical west Africa, subtropical eastern Himalayas, throughout subtropical and tropical Asia into tropical western Pacific and northeastern Australia (Hetterscheid and Ittenbach 1996; Sedayu et al. 2010; Hetterscheid 2012), and 62 species are currently recorded for Thailand (POWO 2023). *Amorphophallus* species usually grow in tropical humid forests, seasonal forests, grass savannahs and secondary forests. Commonly, the members of *Amorphophallus* do not produce their leaves and inflorescences simultaneously (i.e. the inflorescence is produced first and lasts for a short period, and then the leaf is produced, and leaves are only produced for one year for some species.)

During field surveys of plant diversity and medicinal plants in northeastern Thailand in 2019–2022, and our investigations in Sakon Nakhon Province, we collected *Amorphophallus* specimens which were not readily identifiable. After the herbarium specimens and living plants were carefully investigated, we concluded that these were not representatives of any previously-named plant species. Consequently, a new species is described here.

## ﻿Materials and methods

Plant material was collected during field surveys in Khok Si Suphan District, Sakon Nakhon Province in 2019–2022. Morphological observations of the new species were carried out on living plants from the field, as well as on herbarium specimens in BKF and BK. This study consulted the relevant taxonomic literature (such as Gagnepain 1942; Hetterscheid and Ittenbach 1996; Li et al. 2010; Hetterscheid 2012, etc.). The preliminary conservation status of the new species was assessed by applying the criteria (IUCN 2022) given.

## ﻿Taxonomic treatment

### ﻿ Amorphophallus sakonnakhonensis

Taxon classificationPlantaeAlismatalesAraceae

Chatan & Promprom sp. nov.

6D858CB7-05B8-5EBE-AC22-2B5315111D0A

urn:lsid:ipni.org:names:77323169-1

Figs 1 , 2 , 3 , 4

#### Type.

Thailand. Sakon Nakhon Province: Khok Sri Suphan District, northeastern Thailand, 300–320 m elev., 16°59'45.5"N, 104°15'46.9"E, 24 April 2021, *W. Chatan 2897* (holotype: BKF!; isotype: BK!); Khok Sri Suphan District, northeastern Thailand, *W. Chatan 3073* (paratype: BKF!), 16°59'45.5"N, 104°15'49"E, 29 June 2022.

**Figure 1.**
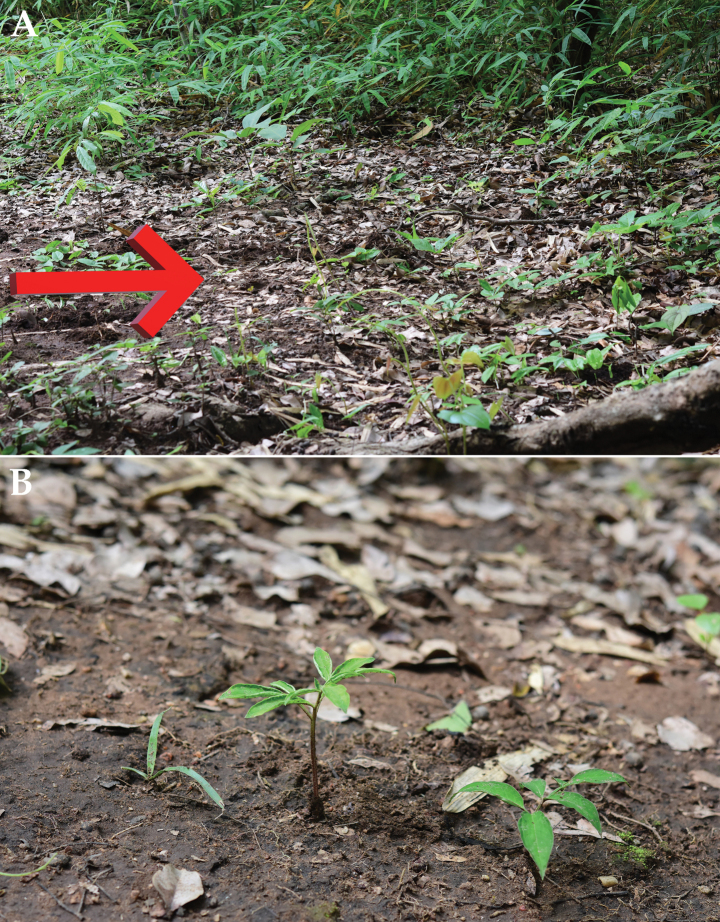
*Amorphophallussakonnakhonensis* Chatan & Promprom. **A** habitat and habit **B** habit.

#### Diagnosis.

*Amorphophallussakonnakhonensis* is most similar to *A.harmandii* Engl. & Gehrm. and *A.linearis* Gagnep. The essential differences with *A.harmandii* are shorter style (ca. 1 mm *vs.* 2–3 mm long); disc-like, slightly smooth surface, concave centre, ca. 0.2 × 0.1 mm stigma *vs.* depressed, shallowly bilobed, ca 0.6 × 1.5 mm; slightly cylindrical, narrower upper part staminate flower zone *vs.* fusiform-conical or lageniform with distinctly dilated basal haft; slightly cylindrical to elongate-fusiform, erect or slightly erect, creamy white appendix *vs.* very narrowly conical, near myosuroid or fusiform conical or slightly sigmoidally curved forward in the lower half, white or greenish yellow.

**Figure 2.**
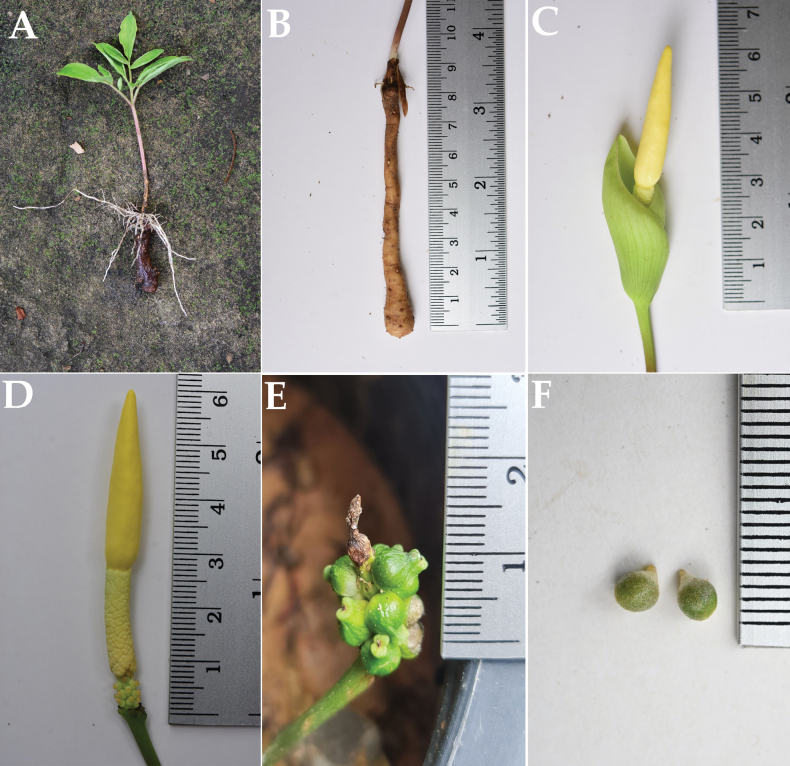
*Amorphophallussakonnakhonensis* Chatan & Promprom. **A** whole plant **B, C** tuber and inflorescence from the same plant **D** spadix **E** infructescence and fruits **F** seeds.

The essential differences with *A.linearis* are disc-like, slightly smooth surface, concave centre, ca. 0.2 × 0.1 mm stigma *vs.* capitate, globose, 1.0–1.3 × 0.9–1.5 mm; elliptic or obovate leaflet *vs.* linear less often lanceolate; 1–3 cm long, creamy white appendix vs. 18–50 cm long, creamy white or green.

**Figure 3.**
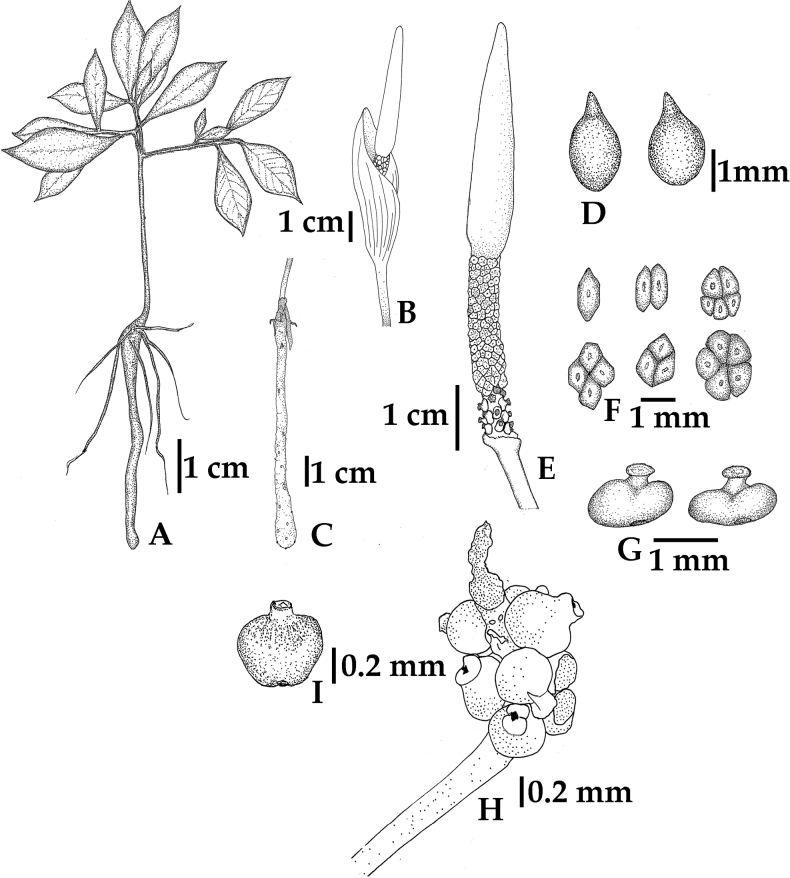
*Amorphophallussakonnakhonensis* Chatan & Promprom. **A** whole plant **B, C** tuber and inflorescence from the same plant **D** seeds **E** spadix **F** staminate flowers **G** pistillate flowers **H** infructescence and fruits **I** fruit.

#### Description.

Perennial herb, narrowly elongated, not branching, 5–10 cm long and 0.4–1.0 cm diam., skin light yellowish to yellowish brown with few or many fibrous lateral roots. Both flowering and leafing plants of small stature, up to 12 cm high. Leaves solitary. Petiole 2.5–2.8 × 0.2–0.3 cm, smooth, white near base and greenish-purple or dark dull red to dark red or rust-coloured at upper part; leaf blade up to 10 cm diam., rachis narrowly winged throughout; leaflet elliptic or obovate, ca. 0.5–3.0 × 0.3–0.5 cm, adaxial side green, abaxial side pale green, apex acuminate. Inflorescence solitary, developed before leaf; peduncle 3.0–5.5 cm long, 2.0–2.5 mm diam., white near base and greenish to greenish-purple at upper part; spathe broadly ovate, both sides dull greenish yellow or creamy white excepting for the deep purple red or bluish purple at base adaxially, 2.0–2.2 × 1.5–1.8– cm, erect, embracing and close to spadix and upper part moving slightly away from spadix during anthesis, smooth on both surfaces, except for lower part verrucate adaxially; spadix subequal or longer than spathe, 2–3 cm long; pistillate and staminate flower zones contiguous, but flowers are slightly distant from each other at connection zone. Pistillate flower zone cylindrical, ca. 1.0 × 0.5 cm, axis green, most flowers slightly distant from each other; ovary depressed, shallow bilobed, ca. 1 × 1 mm, light green; style short, thick, ca. 1 mm long; stigma ca. 0.2 × 0.1 mm, creamy or yellowish, disc-like, surface slightly smooth, centre concave. Staminate flower zone slightly cylindrical, 1.5–2.0 × 0.6–0.8 cm, upper part narrower; staminate flowers fused to a synandrium consisting of 3–5 stamens, creamy or yellowish; filaments united into a short column, ca. 1.0 × 0.8–1.2 mm, whitish or creamy. Appendix slightly cylindrical to elongate-fusiform, 1–3 × 0.8–1.0 cm, erect or slightly erect, creamy white; apex obtuse to slightly acute; surface smooth. Berry green when young and mature, conical to broadly pyriform, shallowly bilobed, ca. 0.5 × 0.5 cm, upper part elongated, odourless, surface verrucate. Seed ellipsoid or ovoid, 2.5–3.5 × 1.0–1.3 mm, apex conical.

#### Flowering and fruiting.

Flowering in May–June and fruiting in June–August.

#### Distribution.

**Figure 4.**
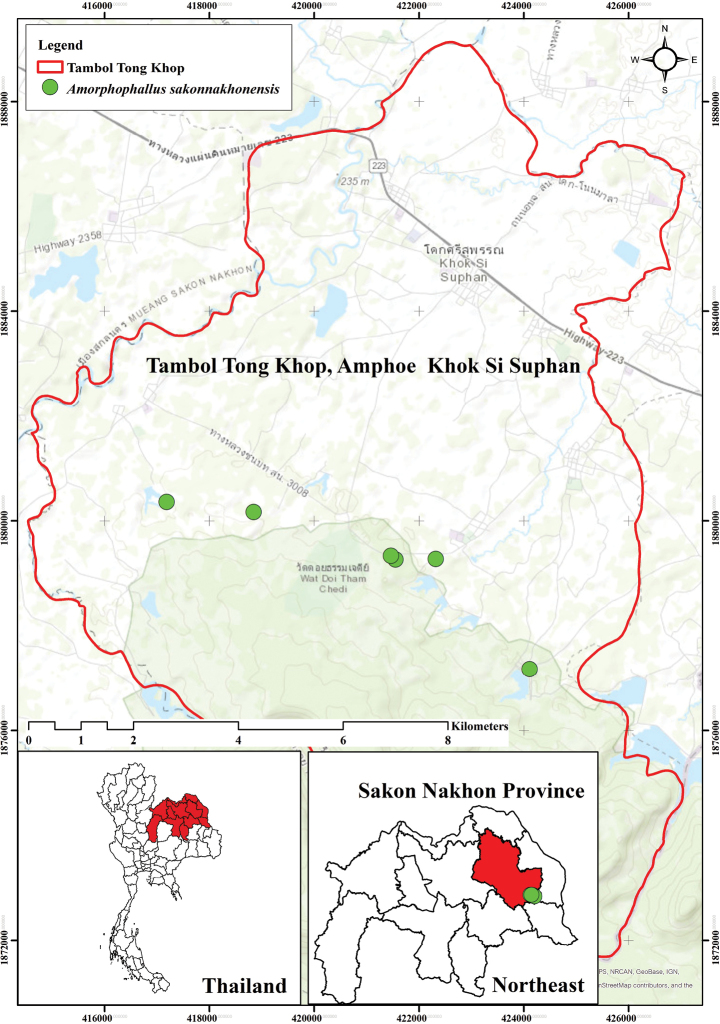
Distribution of *Amorphophallussakonnakhonensis* Chatan & Promprom (green circle).

The new species is endemic to Thailand and is known from only the type locality, Khok Sri Suphan District, Sakon Nakhon Province, northeastern Thailand (Fig. 4).

#### Ethymology.

Specific epithet of *A.sakonnakhonensis* refers to the type locality, Sakon Nakhon Province, northeastern Thailand.

#### Vernacular name.

The Vernacular name of the new species is Buk Noi.

#### Preliminary conservation status.

According to the fieldwork, one population of *Amorphophallussakonnakhonensis* was found at the type locality in Khok Sri Suphan District, Sakon Nakhon Province, northeastern Thailand. However, further explorations are needed for a proper conservation assessment because more information on its distribution and population status is required. Therefore, the species has been preliminarily assigned to Data Deficient (DD) category according to The Guidelines for Using The IUCN Red List Categories and Criteria (IUCN, 2022).

#### Discussion.

*Amorphophallussakonnakhonensis* Chatan & Promprom is similar to *A.harmandii* Engl. & Gehrm. (Engler 1911) and *A.linearis* Gagnep. (Gagnepain 1941), the plants from Thailand and Indochina (Hetterscheid 2012). The new species would be placed in subgenus. Metandrium (Claudel et al. 2017), based on the hypothesis on the phylogenetic affinity of the species, which clearly lies with the *A.harmandii* alliance, close to that species, particularly seen by the near-unique fruits (green, warty). Similarities to *A.harmandii* and *A.linearis* consist of the narrowly elongated tuber, spadix longer than spathe, and contiguous nature of the staminate and pistillate flower zones. The different morphological characters are described below. The main differences of the new species from *A.harmandii* are that it has shorter style; disc-like, slightly smooth surface, concave centre stigma; slightly cylindrical staminate flower zone; slightly cylindrical to elongate-fusiform, erect or slightly erect, creamy white appendix. The new species is also different from *A.linearis* by its disc-like, slightly smooth surface, concave centre stigma; elliptic or obovate leaflet; 1–3 cm long, creamy white appendix. In addition, the size of these organs of the new species are smaller or slightly smaller, including tuber, petiole, leaf blade, peduncle, spathe, spadix, staminate flower zone and appendix. More details of morphological differences amongst *A.sakonnakhonensis*, A.harmandii Engl. & Gehrm. and *A.linearis* Gagnep. are presented in Table 1.

**Table 1.** Morphological differences amongst *Amorphophallussakonnakhonensis*, *A.harmandii* Engl. & Gehrm. and *A.linearis* Gagnep.

Characters	*Amorphophallussakonnakhonensis* Chatan & Promprom	*A.harmandii* Engl. & Gehrm.	*A.linearis* Gagnep.
Tuber	5–10 cm long and 0.4–1.0 cm diam., not branching	ca 40 cm long and 2 cm diam., not or occasionally branching	10–45 cm long and 1–2 cm diam., not or occasionally branching
Petiole	2.5–2.8 × 0.2–0.3 cm	20–80 cm by 4–10 mm	25–85 by 0.5–1.5 cm
Leaf blade	up to 10 cm diam.	36–74 cm diam.	30–80 cm diam.
Leaflet	elliptic or obovate, ca 0.5–3.0 × 0.3–0.5 cm	elliptic, elliptic-lanceolate or lanceolate, 2–20 × 1–7 cm	linear, less often lanceolate, 3–17 × 0.2–1.0 cm
Peduncle	3.0–5.5 cm long, 2.0–2.5 mm diam.	peduncle 6–18 cm long, 3–4 mm diam.	19–55 cm long, 0.3–1 cm diam.
Spathe	broadly ovate, 2.0–2.2 × 1.5–1.8 cm, smooth on both surfaces, except for the base verrucate adaxially	ovate to orbicular, 5–13 × 5–10.5 cm, base densely verrucate, verrucae small adaxially	elongate-triangular or lanceolate, 7–16 × 3.5–6 cm, base within densely clothed with irregular, laterally compressed, ridge-like, sometimes flaky, often confluent, warts, often with short or long hair-like branches
Spadix	2–3 cm long	8–15 cm long	12–57 cm long
Female flowers	slightly distant from each other; style ca. 1 mm long; disc-like, slightly smooth surface, concave centre, ca. 0.2 × 0.1 mm stigma	closed to each other; style 2–3 mm long; depressed, shallowly bilobed, ca 0.6 × 1.5 mm stigma	closed to each other; style 1–2 mm long; capitate globose, 1.0–1.3 × 0.9–1.5 mm stigma
Staminate flower zone size	1.5–2.0 × 0.6–0.8 cm; slightly cylindrical, narrower upper part	3–6 × 1–1.7 cm; fusiform-conical or lageniform with distinctly dilated basal haft	2.5–8 × 0.4–2 cm; conical or slightly lageniform
Appendix	slightly cylindrical to elongate-fusiform; 1–3 × 0.8–1.0 cm, erect or slightly erect; creamy white, never green	very narrowly conical, near myosuroid or fusiform conical or slightly sigmoidally curved forward in the lower half; 5–18 cm × 5–16 mm; creamy white or greenish yellow	myosuroid, base slightly narrowed, 18–50 × 0.5–1.6 cm, at first erect, nodding after anthesis; creamy white or green;

## Supplementary Material

XML Treatment for Amorphophallus sakonnakhonensis

## ﻿Additional information

### Author ORCIDs

Wilawan Promprom https://orcid.org/0000-0001-8821-2267

Wannachai Chatan https://orcid.org/0000-0002-3941-7626

Pattana Pasorn https://orcid.org/0009-0007-3808-7331

Narueset Prasertsri https://orcid.org/0009-0009-1798-1291

Thinnakon Angkahad https://orcid.org/0009-0002-1439-8518

### Data availability

All of the data that support the findings of this study are available in the main text.
